# Robust Preimplantation Genetic Testing of Huntington Disease by Combined Triplet-Primed PCR Analysis of the *HTT* CAG Repeat and Multi-Microsatellite Haplotyping

**DOI:** 10.1038/s41598-019-52769-3

**Published:** 2019-11-11

**Authors:** Mingjue Zhao, Felicia Siew Hong Cheah, Arnold Sia Chye Tan, Mulias Lian, Gui Ping Phang, Anupriya Agarwal, Samuel S. Chong

**Affiliations:** 1 Department of Pediatrics, Yong Loo Lin School of Medicine, National University of Singapore, Singapore, Singapore; 2 Preimplantation Genetic Diagnosis Center, Khoo Teck Puat – National University Children’s Medical Institute, National University Health System, Singapore, Singapore; 3 Clinic for Human Reproduction, Department of Obstetrics and Gynecology, National University Hospital, Singapore, Singapore; 4 Molecular Diagnosis Center and Clinical Cytogenetics Service, Department of Laboratory Medicine, National University Hospital, Singapore, Singapore

**Keywords:** Haplotypes, Genetic testing, Haplotypes, Genetic testing

## Abstract

Huntington disease (HD) is a lethal neurodegenerative disorder caused by expansion of a CAG repeat within the *huntingtin* (*HTT*) gene. Disease prevention can be facilitated by preimplantation genetic testing for this monogenic disorder (PGT-M). We developed a strategy for HD PGT-M, involving whole genome amplification (WGA) followed by combined triplet-primed PCR (TP-PCR) for *HTT* CAG repeat expansion detection and multi-microsatellite marker genotyping for disease haplotype phasing. The strategy was validated and tested pre-clinically in a simulated PGT-M case before clinical application in five cycles of a PGT-M case. The assay reliably and correctly diagnosed all embryos, even where allele dropout (ADO) occurred at the *HTT* CAG repeat locus or at one or more linked markers. Ten of the 27 embryos analyzed were diagnosed as unaffected. Four embryo transfers were performed, two of which involved fresh cycle double embryo transfers and two were frozen-thawed single embryo transfers. Pregnancies were achieved from each of the frozen-thawed single embryo transfers and confirmed to be unaffected by amniocentesis, culminating in live births at term. This strategy enhances diagnostic confidence for PGT-M of HD and can also be employed in situations where disease haplotype phase cannot be established prior to the start of PGT-M.

## Introduction

Huntington disease (HD; OMIM 143100) is an inherited neurodegenerative disease caused by a CAG repeat expansion in exon 1 of the *huntingtin* (*HTT*) gene located on chromosome 4p16.3^1,2^. Affected individuals inherit the expanded allele (≥36 CAGs) from a parent who possesses either an intermediate allele (IA, 27–35 CAGs) or an expanded allele. Clinical symptoms of HD progress gradually, with cognitive loss and psychiatric manifestations appearing first, followed by movement abnormalities, and finally death. Age at onset is inversely correlated with the CAG repeat length^3^, and onset of HD can occur as early as the age of 2 if the expanded allele is larger than 60 (juvenile HD)^4^. Although HD is uncommon (1–2 per 20 000 individuals in populations of western European descent and rarer in other populations^5,6^), the exorable disease progression, and incurable and fatal nature of HD^7^ exert a heavy physical and psychological toll on patients and their families. Consequently, HD patients often develop depression in the early stages^8^, and the suicide rate in patients is at least 5–10 times higher than that in the general population^9–11^. Symptomatic treatment for chorea in HD has been available since 2008^12^ while newer promising antisense oligonucleotide-based therapies that can reduce mutant protein levels lurk on the horizon^13^. However, HD can neither be cured nor its progression delayed. Disease prevention in offspring remains the best option for affected individuals. This can be achieved by preimplantation genetic testing for monogenic disorders (PGT-M), which tests and selectively transfers *in vitro* fertilization (IVF)-derived embryos that are unaffected.

PGT-M for HD began in 1998, employing PCR amplification across the CAG repeat and subsequent capillary electrophoresis for direct expansion mutation detection^14–20^. Due to electrophoretic mobility differences between GC-rich and non GC-rich DNA, allele sizing of GC-rich trinucleotide repeats calculated from size-standard derived amplicon sizes may differ from actual repeat numbers. This can complicate diagnosis in situations where the size of the expanded allele, or one of the normal alleles, in the couple is at the borderline between expanded and non-expanded allele classes, where inaccurate sizing by 1–2 repeats could potentially result in an incorrect disease classification. In addition, immediately downstream of the *HTT* CAG repeat lies a polymorphic CCG repeat stretch, which generally ranges from 7–12 triplets^21^. Some standard PCR protocols generate product that contains both the CAG and adjacent CCG repeats. As a result, the correlation between amplified fragment length and CAG repeat length varies from allele to allele due to the CCG repeat polymorphism. Ideally, standard PCR should amplify only the CAG repeat in order for the product size to be reliably correlated with the CAG repeat length.

Furthermore, like many other trinucleotide repeat disorders, HD exhibits “anticipation” where expanded alleles undergo further expansion upon inter-generational transmission. Due to the GC-rich composition of these repeats, standard PCR may fail to amplify an allele of >100 repeats present in an offspring that is transmitted from a parent with an originally smaller expansion, although such situations are rare^22^. Several ways to improve the performance of standard PCR have been reported^23–26^; however, amplification of large expansions by standard PCR alone is still problematic. Hence, current HD PGT-M assays based on standard PCR rely on observing two normal alleles of different size to unequivocally identify unaffected embryos. This strategy, however, requires that couples must be informative for their normal alleles (i.e. carry normal alleles of different size), and cannot be applied to couples with uninformative normal alleles. This is because in situations where parental normal alleles are of identical size, allele dropout (ADO) of the expanded allele in an affected embryo will yield a result that is indistinguishable from the result of a homozygous normal embryo and thus lead to a misdiagnosis. ADO is defined as the failure to detect either one of the two alleles at a heterozygous locus. Therefore, for couples with uninformative normal alleles, observation of only the normal allele in an embryo is insufficient to make a definitive diagnosis of unaffected status, and it becomes necessary to utilize a PGT-M assay that can detect the expanded allele regardless of its size.

Compared to standard PCR, the triplet primed PCR (TP-PCR) method, which was first described by Warner *et al*.^22^, consistently detects the presence of the expanded allele regardless of its repeat length^27–30^. TP-PCR utilizes three primers – a fluorescently labeled gene-specific 5′ flanking primer, a tailed triplet-primed (TP) primer, and a tail primer. The TP primer consists of five CTGs which allows it to randomly anneal anywhere within the CAG repeat stretch of *HTT* to generate amplicons when paired with the opposing 5′ flanking primer. The tail primer anneals only to the repeat-primed products to generate more product when paired with the opposing 5′ flanking primer. Due to the random annealing of the TP primer within the repeat region, TP-PCR results in a series of amplicons differing in size by 3 bp, with the smallest amplicon containing five CAG repeats and subsequent products increasing in length by one triplet repeat. Expanded *HTT* alleles can be differentiated from normal alleles by the presence of continuous peaks that extend to 36 CAGs and beyond. The major advantage of TP-PCR is that its detection of the expanded allele is not adversely affected by the presence of the much smaller normal allele, which is the major drawback in standard PCR. Additionally, TP-PCR enables precise allele sizing by simple counting of the fluorescent amplicon peaks, thereby avoiding the need for post-capillary electrophoresis mobility correction that is necessary for precise repeat sizing when using standard PCR with internal size standards. To date, however, the TP-PCR method has not been adapted for application to PGT-M of HD, although it has been applied to PGT-M of myotonic dystrophy type 1 and fragile X syndrome^28,31^.

We describe a versatile strategy for HD PGT-M involving the use of TP-PCR for robust detection of the *HTT* CAG repeat expansion^30^, in combination with multiplex-PCR analysis of 13 closely linked microsatellite markers to establish disease haplotype phase^32^. We also report the first clinical application of this strategy for IVF PGT-M of HD in an at-risk couple for which family members of the affected spouse were unavailable for development of a standalone linkage-based PGT-M.

## Results

### Comparison of TP-PCR and standard PCR from single cells and single cell WGA product

**Figure 1:**
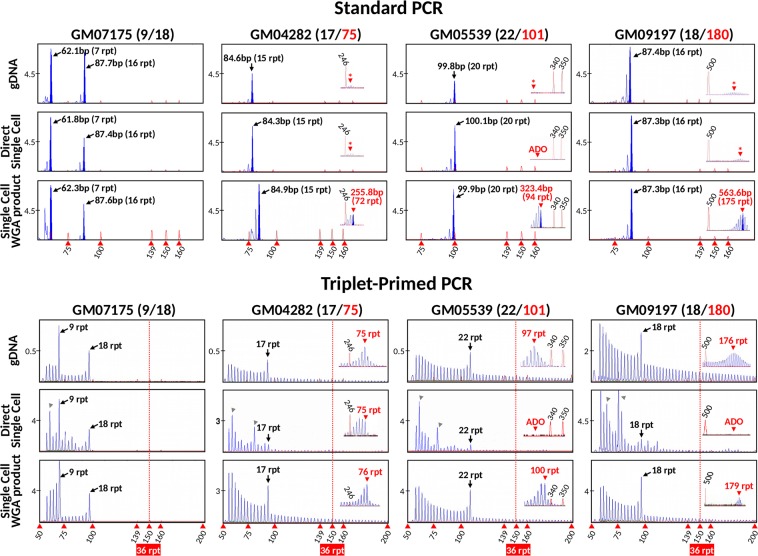
Comparison of standard PCR and TP-PCR for determination of *HTT* CAG alleles. Four cell lines (GM07175, GM04282, GM05539 and GM09197) representing different allele ranges were used and their genotypes are indicated at the top of each column. Standard PCR and TP-PCR were compared at genomic DNA and single cell levels, including direct single cell PCRs and whole genome amplified single cell PCRs. Asterisks denote presence of fluorescent peaks that were too low to be sized by the genetic analyzer. ADO, allele drop-out.

We first compared the sensitivity and reliability of standard PCR and TP-PCR in detecting *HTT* alleles of various repeat sizes from single cells and single cell WGA product from four cell lines GM07175 (heterozygous for normal alleles), GM04282 (17 and 75 CAGs), GM05539 (22 and 101 CAGs) and GM09197 (18 and ~180 CAGs). Standard PCR from both genomic DNA and single cells produced preferential amplification favoring the normal alleles, at the expense of the expanded allele in all three HD-affected cell lines (GM04282, GM05539 and GM09197) such that the expanded allele product was either completely absent or was visible but below the base-pair calling threshold of the genetic analyzer (Fig. 1).

In contrast, TP-PCR from genomic DNA clearly detected both normal and expanded alleles from the four cell lines. Expanded alleles could be easily identified from the continuous stutter peaks extending beyond 150 bp, since TP-PCR product with ≥36 CAG repeats would be indicative of the presence of an expanded allele. TP-PCR from single cells produced irregular peak patterns, which were likely a consequence of the limited DNA template present in a single cell and resulted in greater difficulty in allele sizing, and allele dropout (ADO) of the expanded allele.

When standard PCR and TP-PCR were performed on single cell WGA product, however, detection sensitivity for the expanded allele was significantly improved. Even so, preferential amplification was more pronounced in standard PCR compared to TP-PCR; standard PCR generated much weaker peaks of the expanded allele compared to the distinct peak pattern of the expanded allele observed after TP-PCR. Therefore, TP-PCR is more reliable in detecting expanded alleles and superior to standard PCR as the preferred method for direct expansion mutation detection in whole-genome amplified single cells.

We also noted that the number of repeats obtained from standard PCR products were consistently shorter by ~2 CAG repeats (for normal alleles) and ~3–5 CAG repeats (for expanded alleles) as compared to those determined by counting of TP-PCR fluorescent peaks. In standard PCR, the number of CAG repeats can be determined by subtracting the expected sizes of the repeat-flanking sequences from the fragment size as determined by the GeneMapper software, and dividing the remainder by three. However, the high GC content of these fragments results in accelerated electrophoretic migration and ensuing under-sizing of these fragments compared with average GC content fragments of the same size. The under-sizing of GC-rich fragments in standard PCR can be corrected by using GC-content matched fragments of different lengths to generate a standard curve for mobility correction, which however is tedious and not cost-effective. In contrast, allele sizing from TP-PCR results does not depend on fragment size calculation using internal size standards and is determined by simple counting of the number of peaks after electrophoresis, thus giving a more accurate result. The observed allele sizes agreed with the genotypes of the reference materials as shown previously^30^, demonstrating the accuracy and ease of use of TP-PCR for allele sizing. TP-PCR reliably detects all allele classes, and can precisely size alleles from normal to expanded, with precision sizing limit at ~179 repeats (Fig. 1). The major advantage of TP-PCR compared with standard HD PCR, however, lies neither in its ability to size large expansions more precisely, nor in its non-reliance on mobility correction to accurately size a repeat, but in the fact that it avoids the characteristic preferential amplification of normal over expanded alleles that is observed in standard PCR, thus enabling more confident observation of an expanded allele when it is present.

The reproducibility of TP-PCR in accurately genotyping allele sizes was further evaluated using 10 replicates from each of five whole-genome amplified genomic DNAs of NA20248 and NA20208, which carry 17/36 CAGs and 35/45 CAGs, respectively. All 100 TP-PCR reactions generated the correct genotypes, with neither false positive (detection of ≥36 CAG repeats from the 35-repeat intermediate allele of NA20208) nor false negative (detection of ≤35 CAG repeats from the 36-repeat expanded allele of NA20248) observations (data not shown).

### Assay validation on a simulated PGT-M case

**Figure 2:**
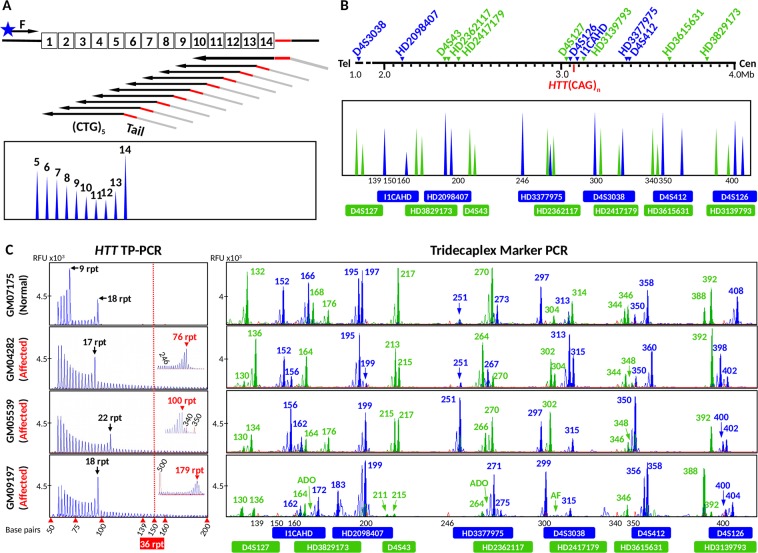
Schematic illustration of the combined PGT-M strategy. Whole genome amplification products from single cells are separately subjected to (**A**) TP-PCR for mutation detection and (**B**) tridecaplex marker PCR for linkage analysis. Electropherograms of *HTT* TP-PCR products and the tridecaplex marker panel PCR products from single cell WGA products of samples are shown displaying a range of alleles (**C**). AF, amplification failure; ADO, allele drop-out.

The TP-PCR assay, in parallel with multiplex-PCR of a highly polymorphic tridecaplex (13-plex) microsatellite marker panel^32^ (Fig. 2) will allow the haplotype phase of the disease/mutant allele to be determined during the PGT-M itself, which is especially useful when there are no family members available for *a priori* haplotype phasing of the disease allele in the affected spouse.

**Figure 3:**
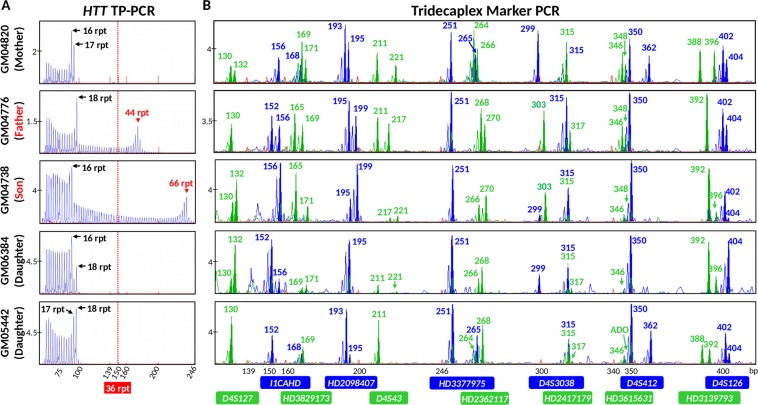
Electropherograms of assay products of parental genomic DNAs and single-cell whole genome amplified products of offspring from an HD family. (**A**) TP-PCR electropherograms. (**B**) Tridecaplex microsatellite marker panel electropherograms. ADO, allele drop-out.

To pre-clinically validate this combined strategy prior to clinical application, we performed a simulated PGT-M cycle by using cell lines from a two-generation HD family. The affected father (GM04776) carries 18 and 44 CAGs, while the mother (GM04820) carries 16 and 17 CAGs (Fig. 3A). Two flanking markers were fully informative in this couple (*HD2362117*, *HD2417179*) while an additional six markers were partially informative (*HD2098407*, *D4S43*, *D4S126*, *I1CAHD*, *HD3615631* and *HD3829173*) (Fig. 3B). To validate this combined strategy for PGT-M of HD, a total of 78 single cells from their three children (28 from GM04738 and 25 each from GM06384 and GM05442) were individually subjected to WGA, and aliquots were separately analyzed by *HTT* CAG repeat TP-PCR and flanking multi-marker PCR. Amplification success at the *HTT* CAG locus was 98.7% (77/78), while allele dropout (ADO) rate was 23.4% (18/77). At the microsatellite loci, amplification success ranged from 89.7% to 100% while ADO rates ranged from 0% to 24.7% (Table 1). ADO rates were within acceptable limits for assays using single cell whole genome amplified DNA as starting template.

**Table 1:** Amplification failure and allele dropout rates at the *HTT* (CAG)_n_ repeat and tridecaplex panel marker loci calculated from GM04738 (n = 28), GM06384 (n = 25), and GM05442 (n = 25) single cells.

Locus	Amplification Failure (AF)	Allele Dropout (ADO)^*^
*D4S3038*	0/78 (0%)	6/53 (11.3%)
*HD2098407*	0/78 (0%)	0/53 (0%)
*D4S43*	0/78 (0%)	5/53 (9.4%)
*HD2362117*	0/78 (0%)	6/78 (7.7%)
*HD2417179*	1/78 (1.3%)	19/77 (24.7%)
*D4S127*	0/78 (0%)	0/53 (0%)
*D4S126*	0/78 (0%)	0/53 (0%)
*HTT(CAG)n*	1/78 (1.3%)	18/77 (23.4%)
*I1CAHD*	0/78 (0%)	4/50 (8%)
*HD3139793*	0/78 (0%)	5/78 (6.4%)
*HD3377975*	1/78 (1.3%)	3/25 (12%)
*D4S412*	0/78 (0%)	3/25 (12%)
*HD3615631*	8/78 (10.3%)	4/49 (8.2%)
*HD3829173*	0/78 (0%)	10/53 (18.9%)

^*^calculated from cell line(s) heterozygous at the locus.

**Figure 4:**
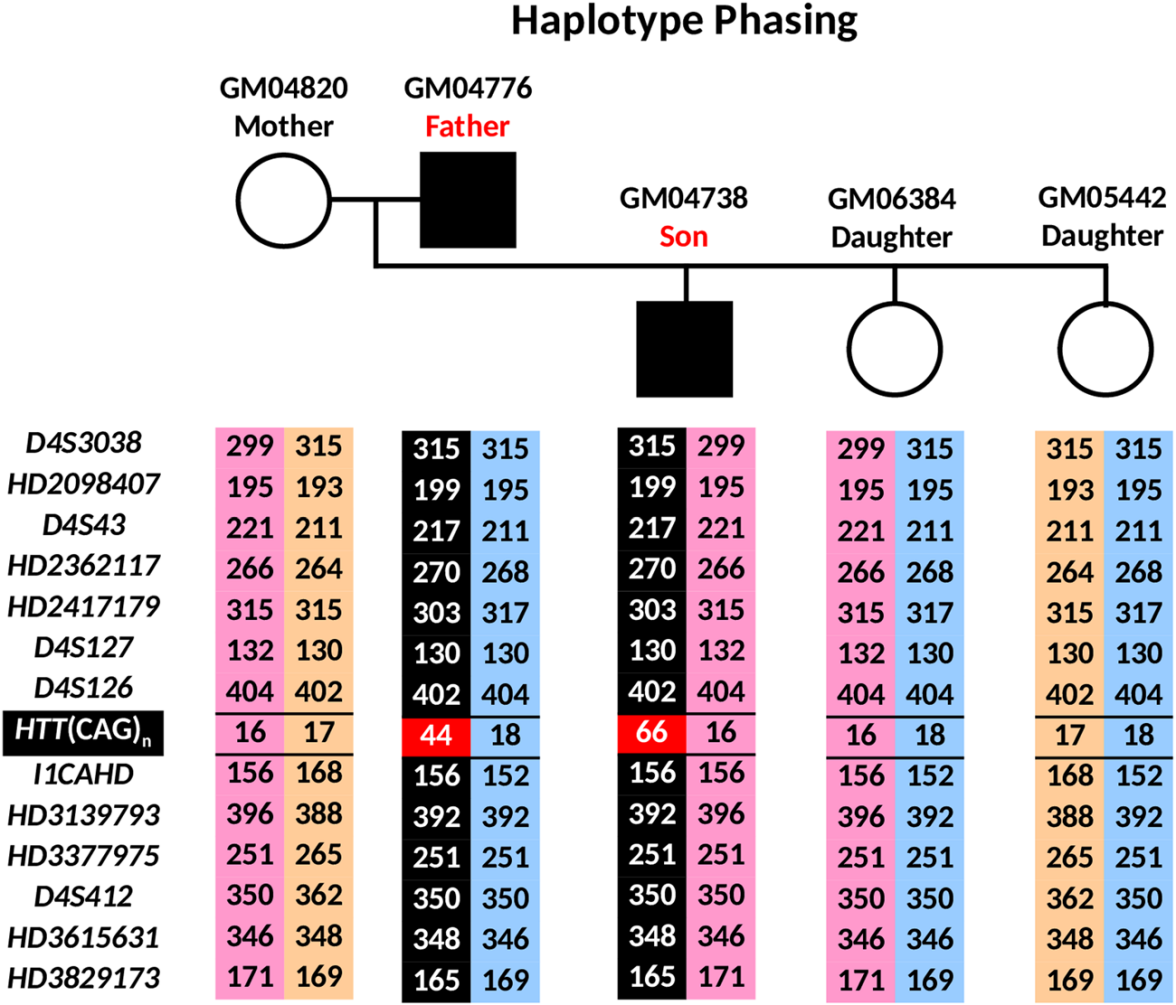
*HTT* (CAG)_n_ genotypes and marker diplotypes of parents and offspring from an HD family. The mutant haplotype is highlighted in black. Affected members are indicated by filled symbols, unaffected members are indicated by empty symbols.

The ADO rate of marker *HD2417179* was observed to be high relative to the other markers even though it is not the largest marker amplicon, and could be due to a bias introduced by WGA. However, based on the other fully informative and three partially informative markers, haplotype phasing of normal and mutant chromosomes could be established for all cells (Fig. 4). Amplification failure at the *HTT* CAG repeat locus was observed in one cell from GM04738, while ADO was observed in 7, 6, and 5 cells from GM04738, GM06384, and GM05442, respectively, which could render the diagnosis uncertain if the PGT-M strategy relied entirely on direct mutation detection. However, based on the haplotype established by the linked markers, normal and mutant chromosomes could be confidently assigned, and an unambiguous diagnosis could be achieved. Nine cells from GM04738 showed CAG repeat sizes (66–76 CAGs) that differed from that of genomic DNA (65 CAGs), reflecting the relative mitotic instability/mosaicism of expanded alleles compared with normal alleles^33^, which nonetheless had no effect on diagnostic accuracy. The combined direct and indirect mutation detection produced an unambiguous “diagnosis” for all single cells of all three cell lines. GM04738 was “diagnosed” as affected while GM06384 and GM05442 were “diagnosed” as unaffected, consistent with their known disease status determined from genomic DNA.

### Clinical IVF PGT-M of HD

**Figure 5:**
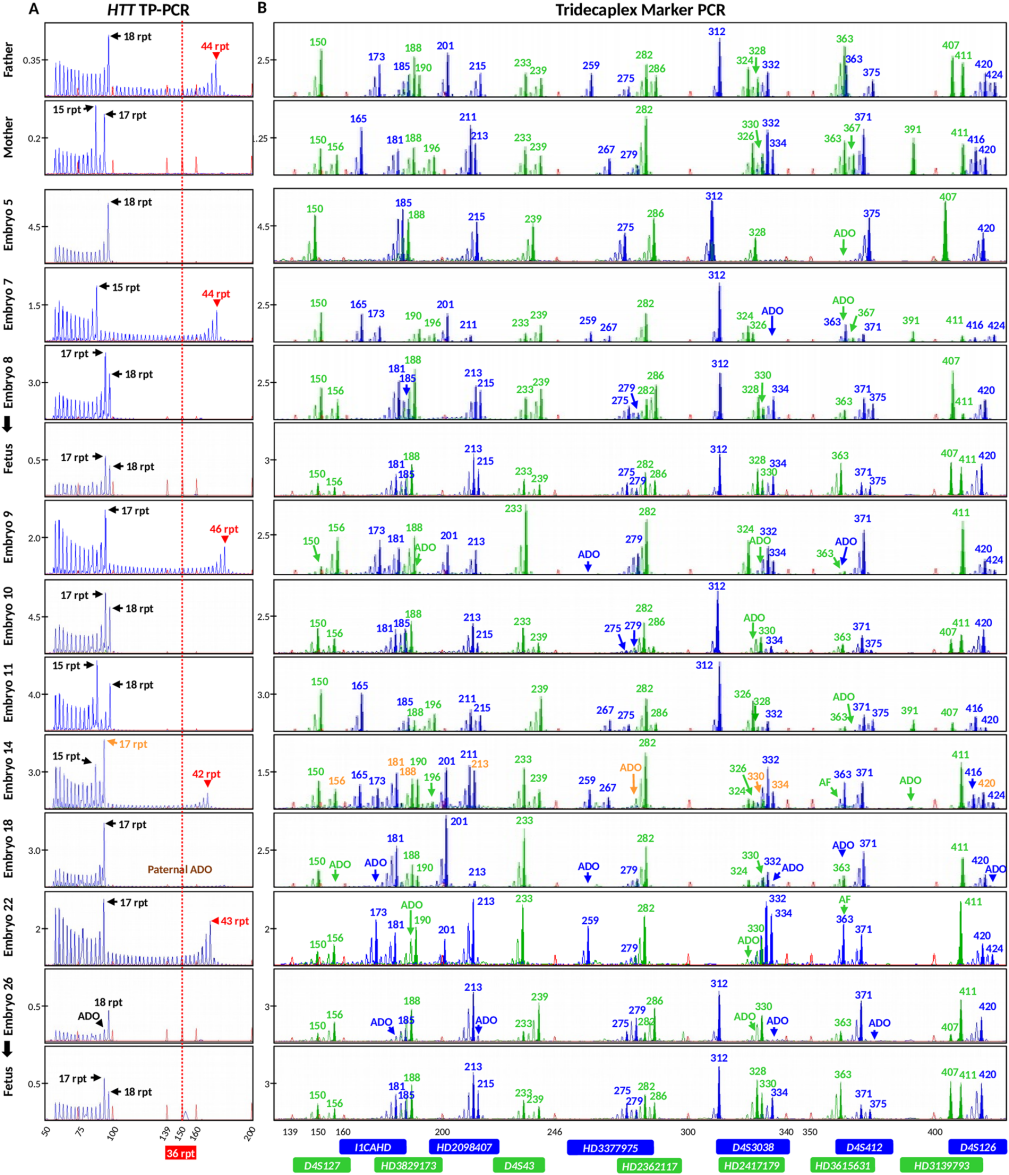
Results of clinical IVF PGT-M case. Electropherograms of (**A**) TP-PCR and (**B**) tridecaplex marker panel PCR are shown for the parental genomic DNAs, blastomere whole genome amplified DNAs, and fetal DNAs from the two successful pregnancies. Embryo 5 displayed the normal paternal haplotype only, while embryo 14 displayed both maternal haplotypes in addition to the affected paternal haplotype. AF, amplification failure; ADO, allele drop-out.

**Figure 6:**
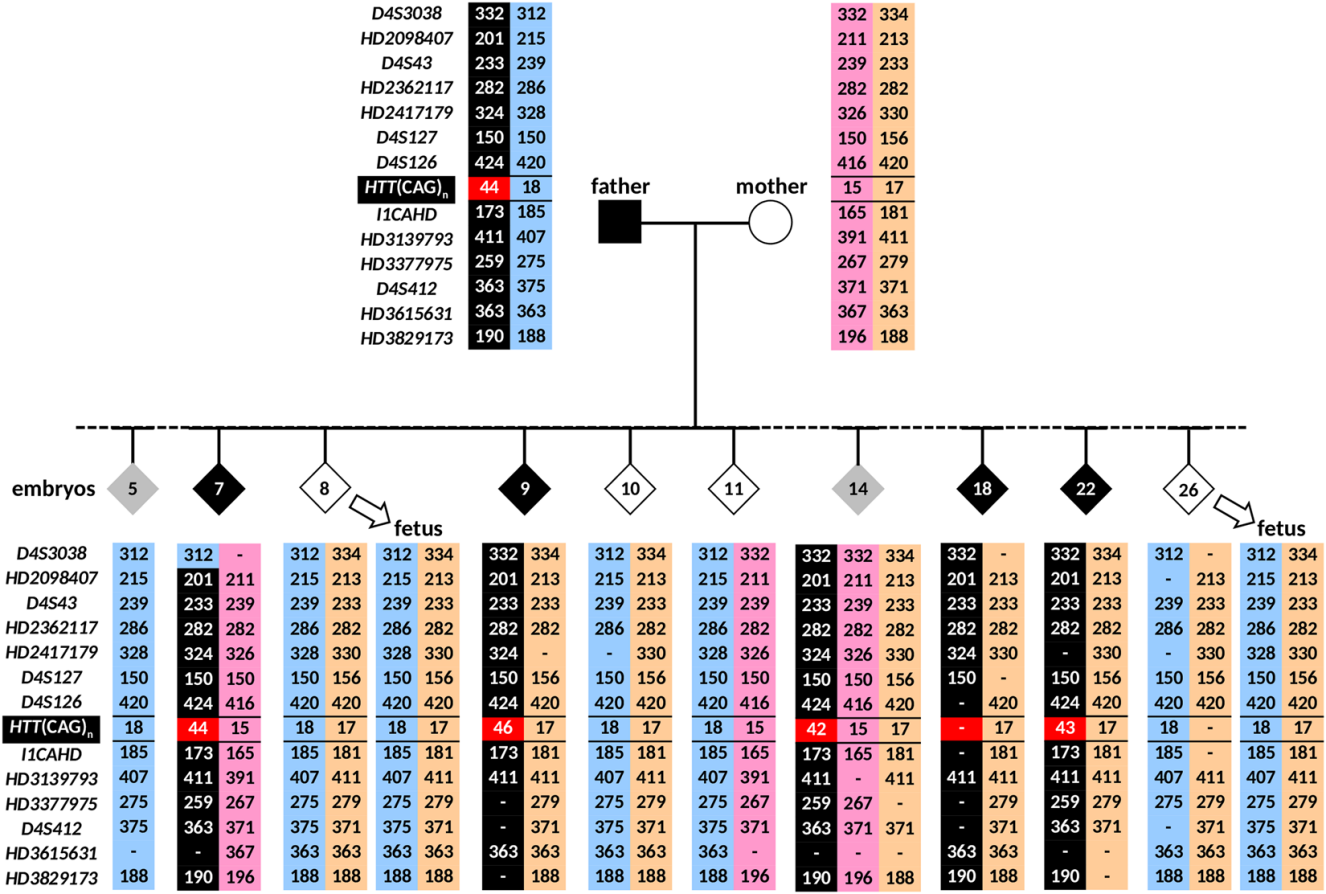
*HTT* (CAG)_n_ genotypes and marker diplotypes of selected embryos and two successful pregnancies from the clinical IVF PGT-M case. The mutant chromosome is highlighted in black. Affected and unaffected embryos are indicated by filled and empty diamond symbols, while embryos with no diagnosis are shown as grey highlighted diamond symbols.

The father was previously genotyped as carrying a normal allele of 18 CAG repeats and an expanded allele of 43 CAG repeats, which we confirmed to within one repeat by TP-PCR (Fig. 5A). The mother was heterozygous for two normal alleles of 15 and 17 CAG repeats (Fig. 5A). Tridecaplex marker analysis identified four fully informative markers (*HD2098407*, *HD2417179*, *I1CAHD* and *HD3377975*), and seven partially informative markers (*D4S3038*, *D4S43*, *HD2362117*, *D4S126*, *HD3139793*, *D4S412*, and *HD3829173*) (Figs 5B and 6).

Standalone indirect linkage-based PGT-M was not possible for this couple, due to the unavailability of any family members of the affected spouse, who are required in order for *a priori* establishment of the disease haplotype phase. Hence, direct detection of the *HTT* CAG repeat was necessary for this couple, and the multi-microsatellite PCR and genotyping was performed on each embryo in parallel to allow disease haplotype phasing during the PGT-M itself.

In total, five IVF PGT-M cycles were performed on this couple, and a summary of all five cycles is shown in Table 2. For blastomeres carrying the expanded allele, slight differences in allele size (40–46 CAGs) were observed, again reflecting increased instability/mosaicism of expanded alleles. ADO was observed at least once for each marker. For one embryo (#22), one of two blastomeres exhibited amplification failure at the *HTT* CAG repeat locus and six markers, and ADO of four additional markers (data not shown). Even so, haplotypes could be correctly assigned, as confirmed by concordance analysis with the second blastomere, which exhibited amplification failure of only one marker and ADO of another two markers. Unambiguous haplotype phasing could be established for all 25 embryos with successful genotyping. A total of 10 embryos were diagnosed as unaffected. Four embryo transfers were performed, of which two were fresh double embryo transfers (cycles 1 and 3), and two were frozen-thaw single embryo transfers (cycles 2 and 5). Both frozen-thaw embryo transfers resulted in pregnancies. Prenatal confirmation of unaffected status was performed by amniocentesis, whereby the same normal CAG repeat sizes observed in the embryo were also observed in the fetus (Figs 5 and 6), and both babies were delivered at term.

**Table 2:** Outcomes of the five IVF-PGT-M cycles.

	Cycle 1	Cycle 2	Cycle 3	Cycle 4	Cycle 5
Oocytes recovered	10	10	10	8	9
Fertilized oocytes	6	6	9	6	5
Embryos biopsied	6	6	7	4	4
Unaffected embryos	2	4	3	0	1
Embryos arrested	—	3	1	—	—
Embryos transferred during the same cycle	2	0	2	0	0
Embryo Frozen & transferred at a subsequent cycle	0	1	0	0	1
Positive hCG	1	1	0	0	1
Pregnancies with fetal heartbeat	0	1	0	0	1
Live-births	0	1	0	0	1

## Discussion

TP-PCR has been shown to provide reliable amplification and detection of repeat expansions regardless of size^25–28,30,34^, and the American College of Medical Genetics and Genomics has indicated that it is the preferred method for genetic testing of HD^35^. Yet, standard PCR is still currently used for direct mutation detection in HD PGT-M^14–20^. One potential pitfall of standard PCR is that a very large expanded allele may fail to amplify or may amplify insufficiently to be detected due to preferential amplification favoring the normal allele (see Fig. 1, for example). Although very large expanded alleles are very rare in HD, the risk of failing to detect a large expanded allele, and thus HD affected status, cannot be ignored especially in the context of PGT-M where the affected spouse carries a large expansion that is at risk of further expansion. Additionally, the CAG repeat sizes calculated from standard PCR fragment lengths may not accurately reflect the actual CAG repeat length, frequently under-estimating by 1–2 repeats due to faster electrophoretic migration of GC-rich DNA compared to non GC-rich DNA (see Fig. 1, for example). In certain situations, such imprecise CAG repeat sizing could lead to ambiguous situations in PGT-M, for example, if the expanded allele in question is 36 or 37 CAG repeats. Methods such as adding additives (Q-solution) and using engineered polymerases (hot start polymerase), which were also employed in our assays, have been shown to improve the performance of standard PCR^24^. Nevertheless, amplification of a large allele in the presence of a much smaller allele is still problematic as shown in Fig. 1. In contrast, this TP-PCR assay has been optimized to reliably amplify and precisely determine CAG repeat sizes from the smallest normal allele up to expanded sizes of ~179 repeats, without any need for migration mobility correction (Fig. 1)^29,30^. Besides, our TP primer design generates the strongest annealing to the furthest position within the repeat region from the opposing flanking primer, therefore generating a tall and distinct last peak representing the largest amplicon to allow clear and easy allele sizing (Figs 1 and 2A), and making it unnecessary to perform extra standard PCR to improve the expansion detection^23,25,26^. Despite its unique ability to amplify and detect repeat expansions regardless of size, TP-PCR has not been widely used in PGT-M of many trinucleotide repeat disorders, including HD. In fact, the concept of TP-PCR can easily be modified for application to other diseases caused by expansions of other repeat motifs.

The combination of expansion mutation detection and disease haplotype tracking is especially beneficial for at-risk couples where *a priori* establishment of disease haplotype phase is not possible due to unavailability of samples from additional family members. For this group of couples, direct expansion detection is indispensable in PGT-M. However, if standalone direct expansion mutation detection is employed, ambiguity may arise from ADO of the expanded/mutant allele in cases where parental normal alleles are uninformative. This ADO risk can be reduced, but not entirely eliminated, by testing directly on blastomeres instead of first subjecting them to WGA which is known to increase ADO rates (Harton *et al*., 2011), and by testing trophectoderm samples instead of blastomeres. Nonetheless, ADO is best overcome by parallel analysis of a highly polymorphic multi-microsatellite panel of closely linked markers^32^ to establish haplotype phase of the disease/mutant allele. For example, haplotype analysis provided a definitive diagnosis when ADO of the affected spouse’s expanded *HTT* allele was observed in embryo 18 of the clinical IVF PGT-M case (Figs 5 and 6). The high marker redundancy and polymorphism of the panel also ensure that haplotyping is not compromised when one or more markers experience amplification failure or ADO. For example, amplification failure of as many as six markers and ADO of an additional four markers was observed in one blastomere of embryo 22, in addition to amplification failure at the *HTT* CAG repeat locus. Nevertheless, the results from the remaining three successfully genotyped markers and the four markers that exhibited ADO was sufficient to establish that the embryo inherited the paternal mutant haplotype and was therefore affected. This diagnosis was confirmed in a second blastomere from the same embryo which produced successful genotyping at the *HTT* CAG repeat locus and 10 marker loci. The high polymorphism of the panel markers also serves to detect exogenous or parental DNA contamination, or even the inheritance of extra parental chromosomes indicative of possible trisomy (e.g. embryo 14, Figs 5 and 6), ensuring higher diagnostic confidence.

As shown throughout all five cycles of the PGT-M case, the combination of the direct mutation detection and the tridecaplex microsatellite marker assay is robust even when applied to whole genome amplified single blastomeres, because haplotype phasing is highly tolerant of random ADO of individual markers when there is ample marker redundancy. Furthermore, this combined assay allows disease/normal haplotypes to be established during the actual PGT-M case, which is useful in cases with no additional affected family members. For instance, in the PGT-M case described above, the disease haplotype (shaded in black in Fig. 6) can be inferred by comparing the alleles of the *HTT* CAG repeat and the informative markers in the parents and in each embryo. Once the disease haplotype phase was determined, the clinical status of each embryo could be subsequently determined. A combined assay not only allows disease/normal haplotype phase determination of each embryo during the PGT-M case itself without the need for extra information from other family members, it also greatly minimizes the “no call” instances caused by ADO of the mutant/expanded *HTT* CAG repeat and is thus diagnostically superior to standalone direct mutation detection.

For couples with available family members, both the TP-PCR and the tridecaplex marker panel will be useful resources for establishing disease haplotype phase before PGT-M begins, although the direct TP-PCR assay can be omitted during PGT-M analysis of the embryos in favor of the more ADO-resilient multi-marker haplotype-based assay. Another significant proportion of couples seeking PGT-M involve individuals who are at risk for HD but do not wish to know their own disease status, in which case direct mutation detection cannot be utilized. Instead, “exclusion PGT-M” is performed to track transmission of the at-risk partner’s at-risk *HTT* haplotype, which requires availability of the at-risk partner’s affected parent to determine the at-risk grandparental haplotype. In this respect, our tridecaplex marker panel was specifically designed and optimized for the *HTT* locus and all 13 markers are highly polymorphic. Therefore, for couples who desire exclusion testing, the tridecaplex microsatellite marker panel will be a useful resource for determining the at-risk individual’s at-risk haplotype, and for tracking the at-risk haplotype during exclusion PGT-M.

Taken together, our optimized TP-PCR assay addresses some limitations in standard PCR and reliably increases diagnostic confidence in PGT-M with minimal steps. This is the first report demonstrating the utility and robustness of TP-PCR of the *HTT* CAG repeat expansion in HD PGT-M, even when day 3 single blastomere analysis is employed.

## Materials and Methods

### Biological samples

DNA samples (NA20248 and NA20208) and cell lines of four unrelated individuals (GM07175, GM04282, GM05539 and GM09197) and five members of an HD family (GM04776, GM04820, GM04738, GM06384 and GM05442) were purchased from the Coriell Cell Repositories (CCR, Camden, New Jersey). Fibroblasts were treated with 0.05% trypsin before single cell isolation and processing. Single lymphoblasts and fibroblasts were obtained and processed as described previously^36^. Single cell whole genome amplification (WGA) was performed using either the REPLI-g Single Cell Kit (Qiagen, Germany) or the illustra GenomiPhi™ V2 DNA Amplification Kit (GE Healthcare, United Kingdom) according to respective manufacturer’s instructions. This study was approved by the Institutional Review Board of the National University of Singapore (07–123E). All methods reported in this manuscript were developed and validated in-house and performed in accordance with ESHRE guidelines for PGT-M.

### TP-PCR, standard PCR, and multiplex microsatellite PCR

TP-PCR, standard PCR, and multiplex microsatellite PCR were performed in separate reactions according to the conditions described below. TP-PCR and subsequent GeneScan analysis were performed as previously described^30^. Briefly, each 25 µl reaction used either 10 ng genomic DNA, 6 µl of lysed and neutralized single cell sample, or 1 µl of whole-genome amplified single cells or 100 pg genomic DNA as template, 1.5x Q-Solution (Qiagen), 1x PCR buffer containing 1.5 mM of MgCl_2_ (Qiagen), 0.2 mM deoxyribonucleic triphosphates (Roche Applied Science, Germany), 2 units of HotStar *Taq* DNA polymerase (Qiagen), 0.5 µM of the F and TAIL primers, and 0.05 µM of TPP primer^30^. When genomic DNA or WGA product was used as template, PCR cycling conditions consisted of an initial polymerase activation step at 95 °C for 15 mins followed by 30 cycles of 98 °C for 45 sec, 63 °C for 1 min, and 72 °C for 2 mins, and a final extension at 72 °C for 5 mins. When single cell samples were used as template, 40 cycles were used.

Standard PCR was performed in a 25 µl reaction containing either 10 ng genomic DNA, 6 µl of lysed and neutralized single cell sample, or 1 µl of single cell WGA product as template, 1 × Q-Solution (Qiagen), 1 × PCR buffer containing 1.5 mM of MgCl_2_ (Qiagen), 0.25 mM deoxyribonucleic triphosphates (Roche Applied Science), 1.25 units of HotStar *Taq* DNA polymerase (Qiagen), and 0.4 µM each of forward^30^ and reverse^37^ primers. When genomic DNA or WGA product was used as template, PCR cycling conditions consisted of an initial polymerase activation step at 95 °C for 15 mins followed by 30 cycles of 98 °C for 45 sec, 60 °C for 1 min, and 72 °C for 2 mins, and a final extension at 72 °C for 5 mins. When single cell samples were used as template, PCR conditions were identical except that a 2 min extension with a 20-second increment per cycle was applied, and number of cycles was increased to 40.

A 1 µl aliquot of the PCR product was mixed with 9 µl of Hi-Di™ formamide and 0.3 μl of GeneScan™ 500 ROX™ dye size standard. The mixtures were denatured at 95 °C for 5 min, cooled to 4 °C, and resolved in a 3130xl Genetic Analyzer (Applied Biosystems) using a 36 cm capillary filled with POP-4™ or POP-7™ polymer. The mixture was electrokinetically injected at 1.2 kV for 18 seconds and electrophoresed for 25 minutes at 60 °C. GeneScan analysis was performed with GeneMapper 4.0 software (Applied Biosystems).

Tridecaplex microsatellite marker PCR analysis was separately performed on genomic DNA or single cell WGA products according to conditions as previously described^32^.

### IVF PGT-M case

The optimized and validated HD PGT-M assay was applied clinically to an at-risk couple. Written informed consent was obtained from the couple to undergo the diagnostic procedure. TP-PCR and tridecaplex marker PCR analysis were separately performed on genomic DNA from the couple to confirm their *HTT* (CAG)_n_ genotypes and to identify informative markers, respectively.

All embryos were generated by intracytoplasmic sperm injection (ICSI) of oocytes. One to three blastomeres were biopsied from each embryo on day three and analyzed separately. Each blastomere was lysed by adding 1.5 µl of 0.6 M potassium hydroxide (KOH, Sigma-Aldrich, United States), heated at 65 °C for 10 minutes, rapidly cooled to 4 °C, and neutralized by 1.5 µl of 0.6 M Tricine (Sigma-Aldrich). Lysed blastomere samples were subjected to WGA using illustra GenomiPhi™ V2 DNA Amplification Kit, and 2 µl of WGA product was used for each TP-PCR or multi-marker PCR assay in 50 µl reactions. The PCR conditions were identical to those described above, and PCR products were analyzed by capillary electrophoresis with either POP-4^TM^ or POP-7^TM^.

## Data Availability

No datasets were generated or analyzed for this study.
